# Attention and Functional Connectivity Among Patients With Early-Stage Subcortical Ischemic Vascular Disease and Alzheimer’s Disease

**DOI:** 10.3389/fnagi.2020.00239

**Published:** 2020-08-13

**Authors:** Min-Chien Tu, Yen-Hsuan Hsu, Jir-Jei Yang, Wen-Hui Huang, Jie Fu Deng, Shih-Yen Lin, Chien-Yuan Lin, Li-Wei Kuo

**Affiliations:** ^1^Department of Neurology, Taichung Tzu Chi Hospital, Buddhist Tzu Chi Medical Foundation, Taichung, Taiwan; ^2^Department of Neurology, School of Medicine, Tzu Chi University, Hualien, Taiwan; ^3^Department of Psychology, National Chung Cheng University, Chiayi, Taiwan; ^4^Center for Innovative Research on Aging Society (CIRAS), National Chung Cheng University, Chiayi, Taiwan; ^5^Department of Medical Imaging, Taichung Tzu Chi Hospital, Buddhist Tzu Chi Medical Foundation, Taichung, Taiwan; ^6^Department of Computer Science, National Chiao Tung University, Hsinchu, Taiwan; ^7^Institute of Biomedical Engineering and Nanomedicine, National Health Research Institutes, Miaoli, Taiwan; ^8^GE Healthcare, Taipei, Taiwan; ^9^Institute of Medical Device and Imaging, National Taiwan University College of Medicine, Taipei, Taiwan

**Keywords:** functional magnetic resonance imaging, regional homogeneity, attention, cognition, sustained attention, divided attention, vigilance

## Abstract

The current study compared attention profiles and functional connectivity of frontal regions in patients with early-stage subcortical ischemic vascular disease (SIVD) and Alzheimer’s disease (AD). Twenty patients with SIVD, 32 patients with AD, and 23 subjects with normal cognition (NC) received cognition and resting-state functional MRI (rs-fMRI) evaluations. The Cognitive Abilities Screening Instrument (CASI) was used to assess global cognition, and simple attention, processing speed, divided attention, and vigilance/sustained attention were evaluated using the Digit Span Forward, Trail Making Test, Symbol Digit Modality Test, and Conners Continuous Performance Test, respectively. Voxel-based regional homogeneity (ReHo) derived from rs-fMRI data was analyzed to identify significant clusters, which were further correlated with attention profiles. Although the patients with SIVD and AD had comparable global cognitive ability, those with SIVD exhibited worse divided attention and vigilance/sustained attention than those with AD. Compared with the NC group, the patients with SIVD exhibited decreased ReHo within the right middle frontal gyrus (MFG) and left anterior cingulate gyrus (ACG), whereas the patients with AD exhibited increased ReHo within the right orbital part of frontal regions. Correlations between these three clusters with attention exhibited distinct patterns according to the dementia subtype, as did attention indices with significance in predicting global cognition. In summary, our study suggested that worse attention performance was associated with functional disconnection within the frontal regions among patients with SIVD than in those with AD. Frontal functional disconnection may underlie the pathogenesis responsible for defective divided attention, vigilance/sustained attention, and notable within-group variations identified in SIVD.

## Introduction

Alzheimer’s disease (AD) and vascular dementia (VaD) have been reported to be the first and second most prevalent types of dementia, accounting for approximately 70 and 17% of all cases of dementia, respectively (Plassman et al., 2007). Subcortical ischemic vascular disease (SIVD) is characterized by small vessel disease and/or lacunes confined within the subcortical structures, and accounts for most cases of VaD (Kalaria, 2016; Dichgans and Leys, 2017). As VaD covers a wide spectrum of pathological changes including ischemia and micro/macrohemorrhage in various locations, SIVD provides an opportunity to explore the impact from vascular burden in the context of pathological and regional constraints (Kalaria, 2016; Dichgans and Leys, 2017). It is important to differentiate patients with SIVD from those with AD to determine their distinct therapeutic strategies (Kalaria, 2016; Dichgans and Leys, 2017). Patients with AD typically report memory and/or other cognitive complaints, in contrast to executive dysfunction exhibited by patients with SIVD owing to impaired fronto-subcortical circuits (Hsu et al., 2016; Tu et al., 2017b). However, pathological reports suggest considerable overlap in subjects primarily classified as having AD/VaD from a clinical perspective (Kalaria, 2016), and thus there has been increased interest in developing accurate and prompt algorithms to identify SIVD. Intuitively, patients with SIVD might be expected to suffer from a greater extent of white matter damage than those with AD; however, both types of dementia exhibit cerebral fiber disconnection to a certain degree to cause cognitive deficits (Absher and Benson, 1993). From a pathological perspective, amyloid plaques and neurofibrillary tangles have been proven to contribute to myelin damage during the very early stage of AD (Tu et al., 2017b; Nasrabady et al., 2018). Disconnection within frontal regions has also been noted in patients with prodromal AD (Neufang et al., 2011). Similarly, vascular burden in SIVD has been shown to trigger Wallerian degeneration, biochemical derangement, and widespread damage to myelin integrity (Lam et al., 2014; Tu et al., 2017b; Liu et al., 2019). Although the myelin damage expected in patients with SIVD is diffuse, previous structural MRI studies have highlighted the role of white matter integrity within the frontal regions. One diffusion tenor imaging study revealed that the genu of the corpus callosum is the most damaged fiber tract in the brain with a SIVD pathology (Liu et al., 2019). Other studies have suggested that both frontal white matter hyperintensities and corresponding microstructural changes are significantly correlated with executive function performance (Hsu et al., 2016; Tu et al., 2017b). In parallel with neuroimaging findings, the theory of disconnection has also been hypothesized to be responsible for cognitive derangement in patients with AD and SIVD in the field of psychology (Delbeuck et al., 2003; Dichgans and Leys, 2017). As an efficient attention network is always required to maintain the implementation of cognition including memory and executive functions (Mccabe et al., 2010), it is therefore possible that attention deficits might exist along with damaged cerebral connectivity (Parks and Madden, 2013; Tu et al., 2018b). Increasing evidence also suggests that frontoparietal attention networks can exhibit dynamic changes in association with task demand (Parks and Madden, 2013). The attention process within frontal regions has been proposed to be of domain-general property, in which activity of neuronal substrates can be allocated regardless of which cognitive domain is in need (Stuss, 2006). Studies investigating attention have proven this concept by highlighting the early emergence of attention deficits and the cognitive impact among demented patients (Silveri et al., 2007; McGuinness et al., 2010; Neufang et al., 2011).

With advances in functional MRI (fMRI), research on cerebral connectivity has extended from structural to functional levels. Using the blood-oxygen-level-dependent (BOLD) fMRI technique, functional connectivity can be delineated by analyzing synchronization among individual cortical regions. Previous fMRI studies of patients with AD have often highlighted signal modifications within hubs in the default mode network (Damoiseaux, 2012). For example, one study identified altered functional activities within the medial prefrontal cortex, bilateral posterior cingulate gyrus/precuneus, and left inferior parietal lobule among patients with AD or mild cognitive impairment (MCI; Zhang et al., 2012). Similarly, five clusters involving the right inferior temporal gyrus, left medial prefrontal gyrus, left anterior cingulate gyrus (ACG), right cuneus, and right middle occipital gyrus have been reported to show decreased functional activity in association with the severity of AD (Wang et al., 2017). Research including patients with SIVD has generally implied a picture representative global dysfunction with or without vulnerability of frontal circuit involvement (Li et al., 2011, 2012; Yi et al., 2012, 2015; Kim et al., 2016; Zhou et al., 2016). Comparisons between patients with AD and SIVD with Pittsburgh compound-B as biomarker basis have shown that patients with SIVD exhibit lower functional connectivity in the medial and superior frontal gyri and anterior insula, whereas patients with AD exhibit defective connectivity within the inferior parietal lobule (Kim et al., 2016). The diversity of these results could be partly explained by dissimilar metric selection and analysis approaches.

Recently, regional homogeneity (ReHo), an important resting-state fMRI (rs-fMRI) metric, has been proposed to be able to effectively quantify synchronization among BOLD time series of a given voxel and its nearest neighboring voxels. Therefore, factors that determine the value of ReHo include both spatial adjacency and functional homogeneity from a time series, and this can provide valuable spatiotemporal information from a neurobiological perspective (Jiang and Zuo, 2016). Increasing evidence suggests that ReHo can be used as an imaging biomarker to monitor and/or identify AD pathology. Altered ReHo signals, mainly involving regions of the default mode network, have been reported to provide distinguishing features with regards to transitions from normal aging to MCI to AD (Bai et al., 2008; Liu et al., 2013). In addition, ReHo has also been shown to significantly correlate with cognitive performance (Zhang et al., 2012; Liu et al., 2013). Although ReHo can provide a network to an extent generally similar to other fMRI metrics, pertinent interpretations of the association between ReHo and severity of AD remain a great challenge (Zhang et al., 2012). Moreover, patients with ostensibly the same cognitive diagnosis can exhibit a mosaic pattern, in which decreases and increases in ReHo values can coexist across multiple brain regions (Liu et al., 2013; Cai et al., 2018), which might be driven by external factors such as treatment response (Cheng et al., 2019). Hence, narrowing the spectrum of demented patients in research is necessary for bridging ReHo knowledge toward clinical relevancy. Although some studies have described functional network properties of patients with SIVD using various fMRI metrics (Li et al., 2011, 2012; Yi et al., 2012, 2015; Kim et al., 2016; Zhou et al., 2016), relatively few studies have investigated ReHo, and even fewer studies have compared patients with SIVD and AD (Kim et al., 2016).

To gain further insight into the brain networks of patients with SIVD and AD, the current study aimed to: (1) compare both attention profiles and ReHo of frontal regions; and (2) investigate the associations between attention performance and ReHo in patients with early-stage SIVD and AD. This study could potentially facilitate the clinical use of rs-fMRI technique and demonstrate the relevance of ReHo on dementia applications.

## Materials and Methods

### Subject Recruitment and Demographic Registry

The patients with dementia were serially recruited from the outpatient clinic in the Dementia Center at our hospital. In this study, a total of 110 cases were initially screened. One patient with recent silent infarct and one with brain tumor were excluded by initial brain MRI scan. Additional 33 cases were excluded because of either missing data (e.g., poor cooperation in completing CPT3 or MRI intolerability; *n* = 20) or other types of dementia (*n* = 13). Therefore, the data of 20 patients with SIVD, 32 patients with AD, and 23 subjects with normal cognition (NC) were used in the following analysis. The inclusion criteria for the patients with SIVD and AD were the same as in our previously published article (Tu et al., 2017b). In brief, both groups of patients exhibited cognitive complaints that interfered with daily activities, and had a Clinical Dementia Rating (CDR) score ranging from 0.5 to 1 (Morris, 1993) and Mini-Mental State Examination (MMSE) score ≤26 (Shyu and Yip, 2001). The diagnosis of AD was based on the National Institute on Aging–Alzheimer’s Association Criteria (McKhann et al., 2011). The diagnosis of SIVD was based on a Hachinski Ischemic Scale score ≥7 (Hachinski et al., 1975) and evidence of subcortical ischemic changes in brain MRI (Erkinjuntti et al., 2000). Only patients with AD and a Hachinski Ischemic Scale score of ≤4 without profound subcortical ischemic changes on brain MRI were recruited into the current study. That is, patients with mixed dementia were excluded.

### Assessment of General Cognition and Dementia Severity

The MMSE is a 30-point questionnaire which usually takes approximately 10 min to complete (Shyu and Yip, 2001). Total scores of the Cognitive Abilities Screening Instrument (CASI) are derived from quantitative assessments of nine cognitive scores, including attention, orientation, short-term memory, long-term memory, language ability, drawing, verbal fluency, abstract thinking, and mental manipulation (Lin et al., 2012). The MMSE and CASI are therefore used to represent global cognition status, where a higher score indicates better cognition. The CDR probes six domains of cognitive and functional performance including memory, orientation, judgment and problem solving, community affairs, home and hobbies, and personal care through a semi-structured interview with the patient and a reliable informant. The rationale for using CDR sum of box to determine the severity of dementia is primarily based on its nonparametric property (Morris, 1993).

### Attention Tests

To evaluate attention performance, the Digit Span (Chen and Chen, 2002), Trail Making Test (Miner and Ferraro, 1998), and Symbol Digit Modality Test of the Wechsler Adult Intelligence Scale (Hinton-Bayre and Geffen, 2005) were administered by a neuropsychologist. The Digit Span consists of two subtests: Forward and Backward. The Forward subtest is used to determine simple attention, whereas the Backward subtest relies more on complex attention, in which working memory and executive function are highly associated (Miloyan et al., 2013). A higher Digit Span score indicates better attention performance. The Trail Making Test, especially part A, is used mainly to assess processing speed (Miner and Ferraro, 1998), with a lower completion time indicating better performance. The Symbol Digit Modality Test is used to measure divided attention (Hinton-Bayre and Geffen, 2005), with a higher number of completions representing better attention status. In addition, the Conners Continuous Performance Test 3 (CPT3; Conners, 2014), a computerized test containing six different blocks, with three sub-blocks each consisting of 20 trials, was also used. It generally takes approximately 20 min to complete this test. During the CPT3, subjects are required to press a button in response to seeing target stimuli that appear on a screen as soon as when any letter appears with the exception of letter X. The interstimuli intervals (ISIs) are 1, 2, and 4 s with a display time of 250 ms. There are six indices in the CPT3 which are designed to measure “inattentiveness” as follows.

1. Detectability: a measure representing the ability to discriminate non-targets (i.e., the letter X) from targets (i.e., all other letters). As it is presented in a reverse-scored manner, a lower value indicates better detectability.
2. Omissions: missed targets. A higher value indicates a higher error rate.
3. Commissions: incorrect responses to non-targets. A higher value indicates a higher error rate.
4. Hit reaction time (HRT): the response speed measured in milliseconds. A longer HRT indicates slowing of information processing speed.
5. HRT standard deviation (HRTSD): the consistency of response speed to targets for the entire test.
6. Variability: the response speed consistency of a respondent.

The CPT3 can also be used to assess vigilance and sustained attention. Assessment of vigilance relies on commission/omission errors and HRT in each ISI change (i.e., HRT_1/2/4_ISI). Assessment of sustained attention relies on commission/omission errors and HRT in each individual block change (BC; i.e., HRT_1/2/3/4/5/6_BC).

### Magnetic Resonance Experiments and Data Analysis

Brain MRI experiments were performed using a 3 T MRI system (Discovery MR750; GE Medical System, Milwaukee, WI, USA) with an eight-channel phased-array head coil. The imaging sequences included three-dimensional T1-weighted imaging (3D-T1), T2 fluid-attenuated inversion recovery imaging (T2-FLAIR), and rs-fMRI. For 3D-T1, the sequence parameters were inversion time (TI) of 450 ms, flip angle of 12°, field-of-view (FOV) of 240 mm, matrix size of 240 × 240, slice thickness of 1 mm, and 160 slices. For T2-FLAIR, the sequence parameters were repetition time (TR) of 12,000 ms, echo time (TE) of 120 ms, TI of 2,200 ms, FOV of 220 mm, matrix size of 384 × 224, thickness of 5 mm, and 21 slices. For rs-fMRI, the echo planar imaging sequence parameters were a TR of 2,500 ms, TE of 30 ms, flip angle of 90°, FOV of 200 mm, matrix size of 64 × 64, thickness of 3 mm, and 47 slices. White matter hyperintensities were rated according to the Fazekas scale from T2-FLAIR sequences, which rates periventricular and deep white matter using a given grade (0–3) depending on the size and confluence of the lesions.

Image quality was assessed independently by two raters (M-CT and S-YL), and the exclusion of datasets with unacceptable image quality was determined by a panel discussion (M-CT, S-YL, and L-WK). The rs-fMRI preprocessing procedures included realignment, slice timing adjustment by using windowed Fourier interpolation, co-registration between echo planar imaging images and T2-weighted images, spatial normalization of T2-weighted images to the Montreal Neurological Institute space (ICBM512 template) using DARTEL (Ashburner, 2007) 24-parameter head motion correction (Friston et al., 1996), and nuisance covariate regression (white matter signals and cerebrospinal fluid signals). The rs-fMRI time series of each voxel was further detrended and band-pass filtered (0.01–0.1 Hz). ReHo measures the similarity between the temporal signals of a given voxel and its neighboring voxels, which we set as the neighboring 26 voxels (Zang et al., 2004). All preprocessing procedures were performed using DPARSF toolbox (Yan and Zang, 2010). The ReHo measurements were analyzed at a voxel-wise level. Significant results were displayed using a referential automated anatomical labeling template atlas (Salvador et al., 2005).

### Statistical Analysis

Analysis of variance (ANOVA) and the *χ*^2^ test were used to detect statistical significance of between-group differences in demographic data, attention tests, and ReHo values. Analysis of covariance (ANCOVA) was used to detect statistical significance by controlling for age and education levels as potential covariates. Spearman’s rank correlation test was used to examine the relationship among attention tests, global cognition (i.e., total CASI score), and ReHo values. Linear regression analysis with a stepwise regression procedure was used to determine the associations between attention indices and global cognition. In the stepwise linear regression analysis, the *p*-values for entry and removal during stepping method criteria were set to be 0.05 and 0.10 by using probability of *F*. The false discovery rate (FDR) was used to examine each individual *p*-value to avoid issues related to multiple comparisons. All statistical tests were performed using SPSS software version 19 (IBM, Armonk, NY, USA). A *p*-value less than 0.05 was considered to be statistically significant.

## Results

### Demographic Data

Table 1 shows the demographic data of the SIVD, AD, and NC groups. There were no significant differences in age, education level, symptom duration, gender, handedness, dementia severity, and global cognition score between the patients with SIVD and AD (*p* = 0.07–0.873). The average symptom durations of the patients with SIVD and AD were 2.5 and 2.9 years, respectively, with mean MMSE scores of 20.1 and 22.6. The patients with SIVD had higher Hachinski and Fazekas scale scores than those with AD (all *p* < 0.001). The NC group had a significantly younger age (*p* = 0.002) and higher education level (*p* = 0.036) than the SIVD group, and a younger age (*p* < 0.001) than the AD group.

**Table 1:** Demographic data comparisons among subjects with SIVD, AD and NC.

	SIVD	AD	NC	
	*N* = 20	*N* = 32	*N* = 23	*P*
Age (years)	75.8 (7.67)	77.3 (6.40)	65.1 (6.97)	***<0.001^bc^***
Education (years)	7.5 (2.81)	8.8 (3.98)	10.2 (3.32)	***0.029^b^***
Duration (years)	2.5 (2.05)	2.9 (1.93)	0.0 (0.0)	***<0.001^bc^***
Gender (Male/Female)	13/7	16/16	11/12	0.258–0.873
Handedness (Right/Left)	19/1	31/1	23/0	0.732
Hachinski Ischemic Scale	9.1 (2.08)	1.7 (1.34)	0.9 (0.89)	***<0.001^ab^***
Fazekas Scale Periventricular white matter hyperintensities	1.9 (0.39)	0.9 (0.53)	0.5 (0.59)	***<0.001^abc^***
Deep white matter hyperintensities	1.9 (0.44)	0.6 (0.60)	0.5 (0.51)	***<0.001^ab^***
Total	3.8 (0.58)	1.5 (0.91)	1.0 (0.92)	***<0.001^abc^***
Clinical Dementia Rating_sum of box	5.1 (4.18)	4.2 (2.71)	0.6 (0.46)	***<0.001^bc^***
The Mini-Mental State Examination	20.1 (5.88)	22.6 (3.92)	27.9 (1.60)	***<0.001^bc^***
Cognitive Abilities Screening Instrument	62.6 (18.38)	70.0 (12.37)	87.7 (4.73)	***<0.001^bc^***

*SIVD, Subcortical ischemic vascular disease; AD, Alzheimer’s disease; NC, Normal Cognition. Data presented as Mean (Standard deviation) unless stated elsewhere. Comparisons were made by using the Analysis of variance (ANOVA) and Chi-square test where appropriate. P-values < 0.05 are boldly italicized. P-values < 0.05 resulting from post hoc Tukey analyses were as follows: ^a^between SIVD and AD, ^b^between SIVD and NC, ^c^between AD and NC*.

### Comparisons of Attention Performance

Table 2 compares attention performance among the subjects with SIVD, AD, and NC. ANOVA showed that the patients with SIVD had more defective subsets of attention tests than those with AD or NC. After controlling for age and education, ANCOVA showed that the SIVD group had worse performance than the AD group in correct subset of the Symbol Digit Modality Test (*p* = 0.026). The patients with SIVD consistently exhibited greater deficits in attention tests than those with AD or NC. Defective performance was shown in the Symbol Digit Modality Test and CPT3. In the Symbol Digit Modality Test, the SIVD group exhibited a lower score in the correct subset than the NC group (*p* < 0.001). The CPT3 test showed that patients with SIVD exhibited a pan-defective pattern of all inattention indices (*p* = 0.002–0.028) except for commission errors, whereas the patients with AD only exhibited defective performance in detectability (*p* = 0.012) and variability (*p* = 0.039). FDR analysis suggested that the patients with SIVD had a lower correct subset of the Symbol Digit Modality Test (*p* < 0.001), greater detectability value (*p* = 0.002), and larger HRTSD (*p* = 0.003) than the subjects with NC.

**Table 2:** Attention test comparisons among the subjects with SIVD, AD and NC.

		SIVD *N* = 20	AD *N* = 32	NC *N* = 23	*P* on ANOVA	*P* onANCOVA	Significance after false discovery rate
Digit forward raw score		8.8 (2.92)	9.8 (2.58)	10.9 (2.17)	***0.043^b^***	0.381	
backward raw score		3.6 (1.91)	4.3 (2.06)	5.8 (2.32)	***0.005^bc^***	0.246	
total		12.5 (4.24)	14.1 (4.22)	16.7 (3.38)	***0.004^b^***	0.198	
Trail making test A		141.9 (109.27)	122.2 (86.88)	59.0 (28.72)	***0.003^bc^***	0.124	
B^†^		283.2 (109.0)	317.5 (196.4)	158.2 (85.91)	***0.003^c^***	0.089	
Symbol Digit Modality Test	Correct	12.8 (8.15)	20.4 (11.70)	36.0 (13.47)	***<0.001^bc^***	***<0.001^AB^***	SIVD < NC
	Error	1.6 (1.55)	1.1 (2.06)	0.8 (1.26)	0.373	0.606	
CPT3	Detectability	−1.6 (1.15)	−1.8 (0.67)	−2.8 (0.66)	***<0.001^bc^***	***0.002^BC^***	SIVD > NC
	Omissions (%)	19.6 (21.98)	11.7 (13.56)	2.2 (2.28)	***0.001^bc^***	***0.015^B^***	
	Commissions (%)	37.6 (21.21)	31.6 (20.7)	25.8 (14.63)	0.144	0.162	
	Hit reaction time (ms)	621.3 (160.30)	557.51 (96.91)	464.9 (84.42)	***<0.001^bc^***	***0.011^B^***	
	Hit reaction time_ standard deviation	208.2 (150.48)	164.6 (52.14)	98.6 (17.81)	***<0.001^bc^***	***0.004^B^***	SIVD > NC
	Variability	66.1 (57.9)	59.8 (40.5)	31.2 (16.54)	***0.012^bc^***	***0.017^BC^***

*CPT3, Conners Continuous Performance Test 3; SIVD, Subcortical ischemic vascular disease; AD, Alzheimer’s disease; NC, Normal Cognition. Data presented as Mean (Standard deviation) unless stated elsewhere. Comparisons were made by using the Analysis of variance (ANOVA) and one-way analysis of covariance (ANCOVA). P-values < 0.05 are boldly italicized. P-values < 0.05 resulting from ANOVA post hoc Tukey analyses were as follows: ^a^between SIVD and AD, ^b^between SIVD and NC, ^c^between AD and NC. P-values < 0.05 resulting from ANCOVA Bonferroni confidence interval adjustments after controlling for age and education were as follows: ^A^between SIVD and AD, ^B^between SIVD and NC, ^C^between AD and NC. ^†^7 SIVD and 20 AD completed this test*.

### CPT3 Analysis of Vigilance and Sustained Attention

**Figure 1:**
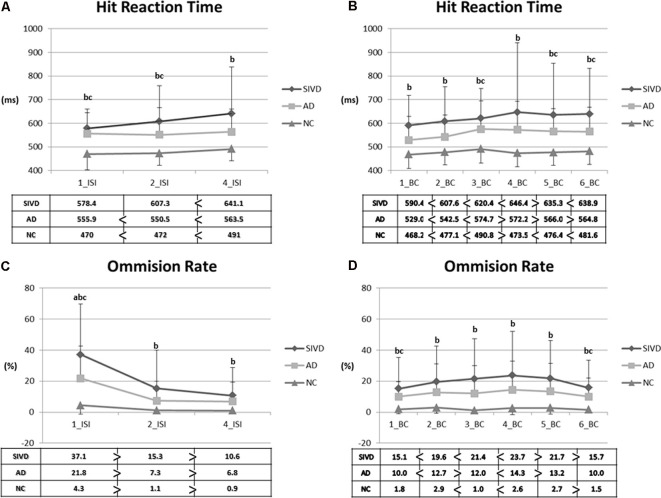
Parameters representing vigilance and sustained attention. **(A)** Hit reaction time (HRT) across interstimulus intervals. **(B)** HRT across blocks. **(C)** Omission rate across interstimulus intervals. **(D)** Omission rate across blocks. 1/2/4_ISI: 1/2/4_seconds_interstimulus interval; 1–6 block change (BC): 1st–6th BCs; SIVD, subcortical ischemic vascular disease; AD, Alzheimer’s disease; NC, normal cognition. Data presented as mean (SD). Comparisons were made by using the analysis of variance (ANOVA) and paired *T*-tests. *P*-values <0.05 resulting from ANOVA *post hoc* Tukey analyses were as follows: a: between SIVD and AD; b: between SIVD and NC; c: between AD and NC.

Given that the patients with SIVD exhibited defective performance in nearly all indices of the CPT3, we further analyzed the index of vigilance and sustained attention in the CPT3. Figure 1 shows the changes in attention indices in relation to different ISIs and BC. Regarding HRT across ISIs, the SIVD group showed a slower response than the NC group across all ISIs (*p* = <0.001–0.001). However, the AD group showed a slower response than the NC group only in 1_ISI (*p* = 0.001) and 2_ISI (*p* = 0.034). No significance was noted in comparisons between the SIVD and AD groups. Regarding HRT across BC, the SIVD group showed a slower response compared with the NC group across all BCs (*p* = <0.001–0.005). In addition, the AD group showed a slower response than the NC group in 3_BC (*p* = 0.016), 5_BC (*p* = 0.043), and 6_BC (*p* = 0.042). After controlling for age and education, significance remained in SIVD/NC HRT comparisons across all ISIs (*p* = 0.025–0.045) and BCs (*p* = 0.010–0.049) except for 4_BC (*p* = 0.080). The only significant difference in SIVD/AD HRT comparisons was in 2_BC (*p* = 0.018). AD/NC HRT comparisons showed no significant differences after adjusting for covariates (*p* = 0.053–1.000). With regard to omission errors, the SIVD group exhibited a higher error rate across all ISIs and BC than the NC group (*p* = <0.001–0.034). Of note, significant differences were noted in omission error rate at 1_ISI among the three groups (i.e., SIVD > AD > NC; *p* = 0.042 in SIVD vs. AD; *p* = 0.012 in AD vs. NC). Other significant between-group differences included higher 1_BC/6_BC error rates in the AD group than in the NC group (*p* = 0.033–0.043). After controlling for age and education, significance remained in SIVD/NC omission error rates in BC_3 to 6 (*p* = 0.013–0.034) and 1_ISI (*p* < 0.001).

### Comparisons of ReHo Values

**Figure 2:**
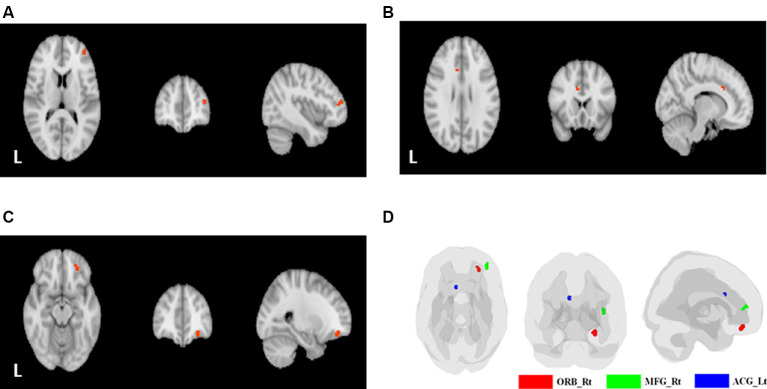
Clusters with significant between-group differences. **(A)** The right middle frontal gyrus (MFG; SIVD < NC). **(B)** The left anterior cingulate gyrus (ACG; SIVD < NC). **(C)** The orbital part of the right superior/middle/inferior frontal gyrus (AD > NC). **(D)** The three significant clusters displayed on a single brain template (green: right MFG; blue: left anterior cingulate gyrus; red: orbital part of the right superior/middle/inferior frontal gyrus).

Table 3 shows the comparisons of voxel-based ReHo after adjusting for covariates including age and education level among the three groups. Note that only the major clusters (100 mm^3^, *p* < 0.001 before FDR correction) were shown. Compared with the NC group, the SIVD group showed two major clusters with decreased mean ReHo values in the right middle frontal gyrus (MFG; peak *p* = 2.3 × 10^−4^; FDR uncorrected) and the left ACG (peak *p* = 2.0 × 10^−4^; FDR uncorrected), whereas the AD group showed one cluster with increased ReHo in the right orbitofrontal cortex (ORB; peak *p* = 1.1 × 10^−4^; FDR uncorrected). No major clusters with significant ReHo differences were found after FDR correction. The aforementioned three clusters are shown in Figure 2.

**Table 3:** Major clusters showing significant differences in regional homogeneity among the subjects with SIVD, AD and NC in voxel-wise cluster analysis (100 mm^3^, *p* < 0.001 uncorrected).

	Regions of interest	Cluster size (mm^3^)	Peak *P*-value	MNI (x y z)			Description	Significance after false discovery rate
SIVD vs. AD	N.S.	−	−				−	−
SIVD vs. NC	Middle frontal gyrus, Rt	270	2.3 × 10^−4^	39	51	15	SIVD < NC	−
	Anterior cingulate gyrus, Lt	135	2.0 × 10^−4^	−12	21	30	SIVD < NC	
AD vs. NC	Superior frontal gyrus, orbital part Middle frontal gyrus, orbital part Inferior frontal gyrus, pars orbitalis, Rt	351	1.1 × 10^−^^4^	24	45	12	AD > NC	−

*SIVD, Subcortical ischemic vascular disease; AD, Alzheimer’s disease; NC, Normal Cognition. Lt, Left; Rt, Right. N.S., non-significant; comparisons were made by using the analysis of covariance (ANCOVA) for controlling age and education*.

### Correlation Analysis Between Attention Performance and ReHo

**Figure 3:**
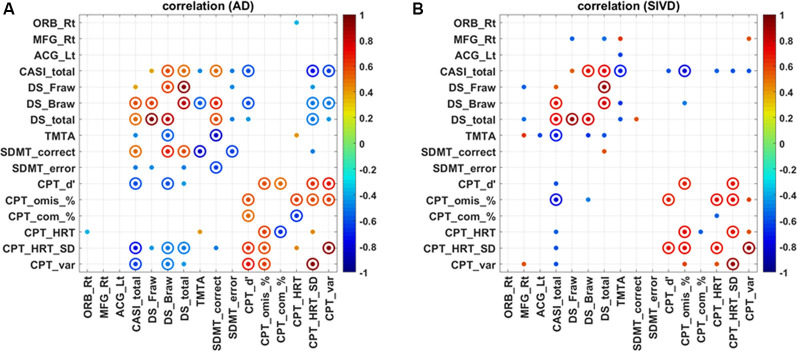
Correlation map regarding regional homogeneity and attention in patients with Alzheimer’s disease **(A)** and subcortical ischemic vascular disease **(B)**. SIVD, subcortical ischemic vascular disease; AD, Alzheimer’s disease; ORB, orbital part of the right superior/middle/inferior frontal gyrus; MFG, middle frontal gyrus; ACG, anterior cingulate gyrus; Rt, right; Lt, left; CASI, Cognitive Abilities Screening Instrument; DS_Fraw/Braw/total, digit forward raw score/backward raw score/total; TMTA, Trail Making Test A; SDMT, Symbol Digit Modality Test; CPT3_d’/omis/com/HRT/HRT_SD/var, Conners Continuous Performance Test 3 detectability/omissions/commissions/HRT/HRT_standard deviation/variability; solid dot: *p* < 0.05; solid circle: *p* < 0.01.

Except for the Trail Making Test B which not all patients completed, the simple Spearman correlations between the mean ReHo values in major clusters and all other attention indices were calculated and are displayed in Figure 3. The correlograms derived from the AD and SIVD groups appeared to be varied. For the AD group, only the right ORB was correlated with HRT of the CPT3 (*ρ* = −0.361; *p* = 0.046), whereas for the SIVD group, the mean ReHo in the right MFG was significantly correlated with the forward score (*ρ* = −0.569; *p* = 0.017) and total score (*ρ* = −0.537; *p* = 0.026) of the Digit Span, Trail Making Test A (*ρ* = 0.608; *p* = 0.027), and variability of the CPT3 (*ρ* = 0.556; *p* = 0.039). In addition, the mean ReHo in the left ACG was correlated with Trail Making Test A (*ρ* = −0.619; *p* = 0.024). There were no significant correlations between the ReHo values in these three clusters and the total CASI score, a representative index of global cognition (*p* = 0.326–0.919).

### Correlations Between Global Cognition and Attention

In the AD group, the total CASI score was significantly correlated with all subsets of the Digit Span (*ρ* = 0.372–549; *p* = 0.002–0.043), Trail Making Test A (*ρ* = −0.453; *p* = 0.013), and all subsets of the Symbol Digit Modality Test (*ρ* = 0.507 in correct; −0.483 in error; *p* = 0.007– 0.012). Regarding CPT3 subsets, the total CASI score was also correlated with detectability, HRTSD, and variability (*ρ* = −0.580 to −0.750; *p* = <0.001–0.001). Stepwise linear regression analysis of the aforementioned significant correlations suggested that HRT_SD (*β* = −0.475, *t* = −3.623, *p* = 0.002), Symbol Digit Modality Test error (*β* = −0.407, *t* = −3.048, *p* = 0.007), and Trail Making Test A (*β* = −0.328, *t* = −2.580, *p* = 0.019) could significantly predict its severity (R^2^ = 0.725, *p* < 0.001). Overall, the model accounted for 72.5% of the variance. The regression model was:

Total CASI score = 99.59 − 0.475 ×HRT_SD−0.407×Symbol Digit Modality Test error −0.328×Trail Making Test A

In the SIVD group, the total CASI score was significantly correlated with all subsets of the Digit Span (*ρ* = 0.532–733; *p* = 0.001–0.028), Trail Making Test A (*ρ* = −0.711; *p* = 0.006), and all subsets of the CPT3 (*ρ* = −0.555 to −0.819; *p* = <0.001–0.032) except for commission errors (*ρ* = −0.183; *p* = 0.468). Stepwise linear regression analysis of the aforementioned significant correlations suggested that total score of the Digit Span (*β* = 0.586, *t* = 4.258, *p* = 0.002) and CPT3 omission errors (*β* = −0.512, *t* = −3.724, *p* = 0.004) could significantly predict its severity (R^2^ = 0.839, *p* < 0.001). Overall, the model accounted for 83.9% of the variance. The regression model was:

Total CASI score = 48.1 + 0.586 × Digit Span total score −0.512×CPT3 omission error

We additionally examined the effect of age, education, and gender on the original linear regression analysis; the linear regression equation results remained unchanged.

## Discussion

The current study indicates that patients with early-stage SIVD and AD exhibit distinct patterns in both attention performance and frontal functional connectivity. Specifically, the SIVD group exhibited worse performance in divided attention and vigilance/sustained attention than the AD group. In contrast to the significantly increased mean ReHo value in the right ORB in the AD group, significant decreases in ReHo values in the right MFG and left ACG were noted in the SIVD group. Distinct patterns of correlations between the mean ReHo values in the three major clusters and the attention indices were observed. Further linear analysis derived from correlograms also indicated different attention indices in predicting global cognition in the SIVD and AD groups.

### Attention Profiles of the Patients With AD and SIVD

As evidenced by the Symbol Digit Modality Test, our results showed that the SIVD group had worse divided attention than the AD and NC groups. This finding reflects that limited cognitive capacity is one of the cognitive features among patients with SIVD. We also observed that the capacity to simultaneously integrate multi-stimuli was limited among the patients with SIVD, who often experienced cognitive overload during casual conversations and/or cognitive tests. These findings are compatible with the cognitive profiles identified by Scherr et al., in which divided attention (Scherr et al., 2014) and speed of cognition (Jokinen et al., 2009; Scherr et al., 2014; Richards et al., 2019) were notably affected. Consistently, more defective parameters involving HRT of the CPT3 were also observed in the patients with SIVD than in those with AD. Although, we did not observe a foreperiod effect (i.e., decreasing HRT with increasing ISI) among the three groups, the curve of HRT in relation to ISIs from the SIVD group appeared to be distinct from the other two groups (Stuss, 2006). This deficit is primarily thought to be a deficit of decreased monitoring, that is, impaired vigilance (Stuss, 2006). Between-group comparisons showed statistically significant deficit in HRT and omission errors across all ISIs among the patients with SIVD, in contrast to the defective performance of the patients with AD which was confined to 1_ISI and 2_ISI only. Of note, there was a discernible 1_ISI omission error rate across the three groups. That patients with SIVD made errors on rapid eminent visual stimuli and failed to exhibit readiness in response to variable ISIs signifies a decrease in vigilance (Conners, 2014). In contrast, dynamic performances across BCs in the AD group were more stable, with major defective points confined to the latter blocks. Therefore, the AD patients appeared to have sustained attention deficits, but to a lesser degree and severity than those with SIVD. Accordingly, deficits in divided attention, vigilance, and sustained attention appear to be critical cognitive features in patients with SIVD. It is also interesting that our MRI-based analysis identified greater variations in HRT in the SIVD group compared with the other two groups, which confirms the association between small vessel disease and a high level of within-group variation in view of the reaction time measures (Richards et al., 2019).

### Distinct Degree and Spatial Coordinates of Frontal Functional Connectivity in the Patients With AD and SIVD

Aside from more attention deficits, the patients with SIVD also showed distinct alterations in the degree of functional connectivity and spatial coordinates to those with AD. Of note, the patients with SIVD and AD showed opposite degrees of ReHo alterations in our study. The decrease in ReHo in the SIVD group indicated an ongoing profound disconnection process. Intracranial atherosclerotic changes may hinder the amount and efficiency of cerebral blood flow delivery, leading to decreases in the amplitude and/or phase discordance of BOLD signals within individual neuron clusters. Impaired cerebral perfusion is consequently reflected by low ReHo values. This hypothesis may also explain the lag in CPT3 responses that were most notable at 1_ISI, in which the patients with SIVD needed a lengthy period to deal with challenges after the preceding visual clues. In contrast, an increase in ReHo and/or BOLD signal values in patients with AD have frequently been linked with a hypothetical compensatory process (Peraza et al., 2016), in which increased connectivity at the very early stages is conceptualized to maintain the implementation of cognition during a neurodegenerative process. The compensatory hypothesis is supported by a study that recruited patients with AD undergoing treatment, in whom a decrease in ReHo was observed in line with improving cognition (Cheng et al., 2019). Similar findings have also been reported in previous studies, including decreased functional synchrony along with degenerative pathology (Hedden et al., 2009; Sheline et al., 2010; Wang et al., 2017), and better memory capacity being related to higher ReHo values (Zhang et al., 2012). However, other studies have reported different or even opposite results. A previous study incorporating PET with fMRI revealed that normal elderly with positive amyloid load exhibited lower functional connectivity than those without amyloid load (Hedden et al., 2009). Another study also reported a mosaic pattern regarding functional connectivity, in which age-related alterations were observed to a greater degree in the anterior than the posterior default mode network (Jones et al., 2011). Taken together, these diverse findings suggest that it might be too early to conclude an overall effect regarding changes in ReHo value, as dynamics of cerebral blood flow could be highly affected by a wide spectrum of degenerative states and possible interregional reciprocal changes.

Another important finding of this study is the clinical relevance regarding spatial distribution of the major clusters that we identified. Both the right MFG and left ACG are strongly involved with the attention network. It has been reported that the ACG is associated with conflict processing, response selection, and movement execution (Fan et al., 2007), and that the right MFG is located within the broad functional connectomes centered by the ACG (Fan et al., 2007). We, therefore, hypothesize that lower ReHo values in the right MFG and left ACG may be partly related to the pronounced slow responses with errors in the CPT3 made by the patients with SIVD. Based on the inverse correlation between mean ReHo values in the left ACG and the Trail Making Test A, lower ReHo values were linked with dampened neuronal activity that may consequently affect attention. This hypothesis could be further extrapolated to the mechanisms contributing to apathy (Tu et al., 2017a), executive dysfunction (Hsu et al., 2016), and gait problems (Tu et al., 2018a) commonly identified in patients with SIVD. An increase in ReHo values within the right ORB among the patients with AD provided an interesting parallel compared with the aforementioned distinct findings among the patients with SIVD. An animal study highlighted the role of the ORB in comparison between currently and previously attended information, compared with the ACG primarily in directing action after updating new information (Hunt et al., 2018). The results of our omission rate analysis of the CPT3 showed that the patients with AD had higher error rates than the subjects with NC in 1_ISI but not in those with longer ISIs. This indicates the possibility that a longer preparation period after visual stimuli could help the patients with AD with regards to attention control to make a correct response.

Our correlograms also revealed significantly inverse correlations between ReHo within the right ORB and HRT of the CPT3. ReHo alterations in the right ORB may reflect a state of compensatory response, as insults in the superior medial frontal regions have been commonly associated with slowing of reaction time in a variety of psychological experiments (Stuss, 2006). Aside from the three clusters currently reported and hubs of the default network, the neurovascular signal changes in other regions have also been addressed, such as the lingual gyri, inferior frontal gyrus, and fusiform gyrus in patients with AD (Peraza et al., 2016), and the right thalamus, left caudate nucleus, cingulate, bilateral superior temporal, and left ventral subcallosal gyri in patients with SIVD (Yang et al., 2002). The spatial variations could be partly caused by differences in imaging modalities, processing, and cohort demographics.

### Neuronal Correlates and Cognitive Impact of Attention

The correlograms varied according to the types of dementia, which suggests several important findings. First, discernible patterns of attention profiles were further visualized by three clusters that were inherently different in terms of their degree and locations. Second, ReHo values within the right ORB, right MFG, and left ACG showed relatively convergent associations toward attention profiles rather than global cognition. Finally, the results showed the cognitive impact from individual attention components, including that weighting and predictive factors varied according to the subtype of dementia. In contrast to the divergent correlations between global cognition and attention, the three clusters demonstrated pairs of correlations confined within attention performance. This highlights the reliability of our rs-fMRI data on the basis of reasonable predicting factors for global cognition with considerable explanatory variance.

Intriguingly, there is a growing evidence base suggesting the value of cognitive rehabilitation in reducing both physical and functional disability of demented patients (Bahar-Fuchs et al., 2013; Zucchella et al., 2018). As cognitive rehabilitation highlights a goal-oriented approach to maintain potentially achievable functional status (Bahar-Fuchs et al., 2013), evaluation of attention profiles and functional connectivity may aid in optimizing therapeutic approaches. In addition, the goal and needs are often dynamically tailored along with dementia stage. To monitor the effect of either restorative or compensatory approaches (Bahar-Fuchs et al., 2013; Zucchella et al., 2018), attention and ReHo indices could serve as potential markers in aiding such personalized intervention strategy.

The strengths of this study include the comprehensive exploration of attention components in addition to voxel-based ReHo analysis of rs-fMRI. The distinct patterns of attention profiles and changes in ReHo values may provide valuable information to characterize dementia subtypes and assist in the differential diagnosis. In addition, the different weighting of individual attention components for global cognition in the patients with SIVD and AD also elucidated disease-specific cognitive processes. The main limitation of this study is the relatively small number of cases, which may have contributed to statistical bias. In addition, as a considerable portion of AD patients are reported having systemic and/or cerebral vascular pathologies, future larger-scale studies incorporating other biomarkers such as APOE genotyping or amyloid scan are warranted to verify the current hypothesis (Lee et al., 2020). Even though, we defined main attention profiles according to each individual measure, there was no uniform agreement in terms of these individual attention processes as an overlapping effect may have existed. As this study focused on attention performance, we were unable to address the effect of other cognitive domains that may have existed. Despite our best efforts to collect patients with an early stage of dementia, the variations in HRT in the patients with SIVD remained greater than in those with AD, suggesting the possibility of heterogeneity and/or interpersonal variability in the current SIVD cohort. Such variability may also have mitigated the statistical robustness, and therefore some meaningful between-group differences in the current data may have been missed. Finally, as an inevitable challenge for most rs-fMRI studies, the rs-fMRI and cognitive tests were usually conducted at different times, causing uncertainty to fit the assumption of constant temporal functional interactions throughout the resting state (Allen et al., 2014). However, it could be an even greater challenge to reliably decouple dynamic cerebral functional changes with current cognitive experiments, as both traditional attention tests and/or the CPT3 are a complex paradigm of the attention process. Therefore, ReHo remains a potentially useful MRI metric and may provide regional-specific insights of targeted cognitive processes.

## Conclusion

This study showed that the patients with SIVD exhibited worse divided attention and vigilance/sustained attention than those with AD. A greater extent of functional disconnection within the frontal region in the patients with SIVD compared with those with AD supports the limited capacity of monitoring and dynamic allocation of neuronal activity. Furthermore, patterns of ReHo–attention correlograms varied by dementia subtype with regard to significant correlations with the right ORB, right MFG, and left ACG. These findings highlight the fact that patients with SIVD and AD exhibit distinct attention profiles and frontal functional connectivity responsible for individual attention components.

## Data Availability Statement

The datasets generated for this study are available on request to the corresponding author.

## Ethics Statement

The studies involving human participants were reviewed and approved by The Research Ethics Committee of the Taichung Tzu Chi Hospital, Buddhist Tzu Chi Medical Foundation (REC 106-09). The patients/participants provided their written informed consent to participate in this study.

## Author Contributions

M-CT: patient recruitment and clinical examination. M-CT and L-WK: study concept and design, analysis, interpretation, and drafting the article. Y-HH, W-HH, and JD: neuropsychological test assessment and interpretation. S-YL: graph design, analysis, and drafting the article. J-JY and C-YL: neuroimaging processing. All authors contributed to the article and approved the submitted version.

## Conflict of Interest

The authors declare that the research was conducted in the absence of any commercial or financial relationships that could be construed as a potential conflict of interest.
